# Long-term outcome of bicuspid aortic valve disease

**DOI:** 10.1093/ehjci/jead312

**Published:** 2023-11-15

**Authors:** Julia Aschauer, Robert Zilberszac, Andreas Gleiss, Christian Colizzi, Thomas Binder, Piergiorgio Bruno, Günther Laufer, Massimo Massetti, Harald Gabriel, Raphael Rosenhek

**Affiliations:** Department of Cardiology, Vienna General Hospital, Medical University of Vienna, Waehringer Guertel 18-20, Vienna 1090, Austria; Department of Cardiology, Vienna General Hospital, Medical University of Vienna, Waehringer Guertel 18-20, Vienna 1090, Austria; Center for Medical Data Science, Institute of Clinical Biometrics, Medical University of Vienna, Vienna, Austria; Institute of Cardiology, Catholic University of Sacred Heart, Rome, Italy; Department of Cardiology, Vienna General Hospital, Medical University of Vienna, Waehringer Guertel 18-20, Vienna 1090, Austria; Institute of Cardiology, Catholic University of Sacred Heart, Rome, Italy; Department of Cardiac Surgery, Medical University of Vienna, Vienna, Austria; Institute of Cardiology, Catholic University of Sacred Heart, Rome, Italy; Department of Cardiology, Vienna General Hospital, Medical University of Vienna, Waehringer Guertel 18-20, Vienna 1090, Austria; Department of Cardiology, Vienna General Hospital, Medical University of Vienna, Waehringer Guertel 18-20, Vienna 1090, Austria

**Keywords:** bicuspid aortic valve, aortic stenosis, aortic regurgitation

## Abstract

**Aims:**

Bicuspid aortic valve (BAV) is a common congenital condition that is frequently associated with aortic stenosis (AS) and aortic regurgitation (AR), as well as aortic aneurysms, but specific outcome data are scarce. The present study sought to assess outcomes in a large cohort of consecutive patients with BAV.

**Methods and results:**

A total of 581 consecutive patients (median age 29 years, 157 female) with BAV were included in the study and followed prospectively in a heart valve clinic follow-up programme. The overall survival rate after 10 years was 94.5%. During follow-up, 158 patients developed an indication for surgery. Event-free survival rates were 97%, 94%, 87%, and 73% at 1, 2, 5, and 10 years, respectively. In the multivariable analysis, event rates were independently predicted by AS [subdistribution hazard ratio (SHR) 2.3 per degree of severity], AR (SHR 1.5 per degree of severity), baseline aortic dilatation ≥ 40 mm (SHR 1.9), and age (SHR 1.3) (*P* < 0.001).

**Conclusion:**

BAV disease is associated with a high rate of cardiac events, but state-of-the-art care results in good survival with low rates of infective endocarditis, aortic dissection, and sudden death. Incremental degrees of AS and regurgitation, the presence of aortic dilatation, and age are predictive of cardiac events.

## Graphical Abstract

**Graphical Abstract:**
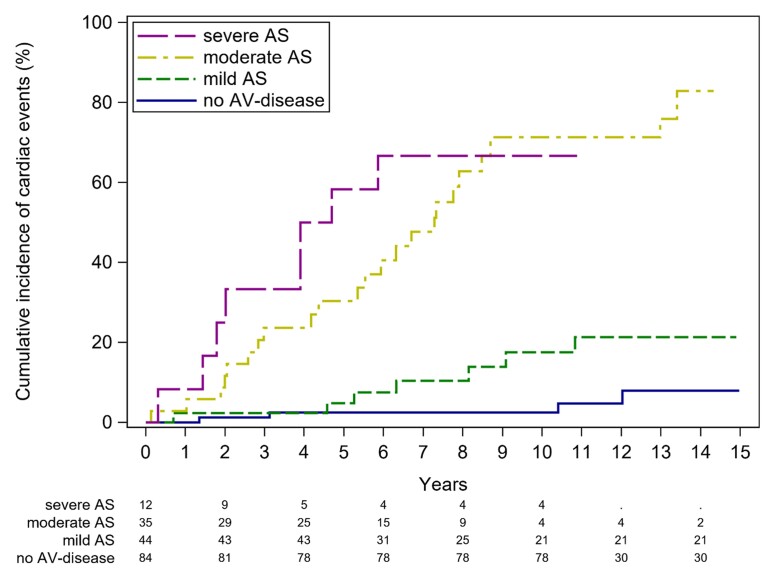

## Introduction

Bicuspid aortic valve (BAV) is the most common congenital cardiac anomaly with a prevalence between 0.5% and 2% in the general population.^1^ BAV is associated with aortic stenosis (AS), aortic regurgitation (AR), and aortic dilatation.^2^ It is assumed that almost 50% of isolated severe AS cases requiring surgery have an underlying BAV pathology.^3^

Patients with BAV are at risk of developing an indication for surgery due to severe aortic valve disease or progression of aortic dilatation to aortic aneurysm and/or dissection. The likelihood of presenting with AR is higher in younger patients with BAV, while older patients present more frequently with AS. Nonetheless, presentation with severe AS occurs at a younger age in patients with BAV when compared with patients with tricuspid AV stenosis.^3^

The prognostic value of the severity of aortic valve disease (stenosis and regurgitation) and of age on the natural history of BAV disease is scarcely defined,^4^ while limited data are available on aortic dilatation.^5^

The present study sought to assess the natural history of a large cohort of consecutive patients with BAV followed in the setting of a dedicated heart valve clinic (HVC) in a tertiary centre. In particular, the aim was to assess the predictive value of AR severity, AS severity, and baseline aortic dilatation on clinical outcome and cardiovascular events. Furthermore, it was sought to assess the impact of age on outcome.

## Methods

### Patient population

All consecutive asymptomatic patients with a confirmed diagnosis of BAV disease that was seen in our HVC between 1995 and 2012 were included in the study. Exclusion criteria were symptoms related to AS or AR, impaired left ventricular ejection fraction (LVEF < 50%), a history of previous surgery on the aortic valve or the ascending aorta, and additional haemodynamically significant valve lesions (moderate or severe).

According to these criteria, 581 consecutive patients were identified. The study protocol was approved by the ethics committee of the Medical University of Vienna, and written informed consent was not demanded due to the observational study design.

### Clinical data

At study entry, clinical data were recorded as follows: demographic data including age and gender; cardiac rhythm; and cardiovascular risk profiles including the history of hypercholesterolaemia (total cholesterol > 200 mg/dL or patient under lipid-lowering therapy), arterial hypertension (blood pressure > 140/90 mm Hg on the basis of the average of repeated measurements), coronary artery disease (CAD) (history of myocardial infarction, angioplasty, coronary artery bypass grafting, or angiographically documented coronary artery stenosis), and diabetes mellitus. Information on concomitant statin, beta-blocker, angiotensin-converting enzyme inhibitor, angiotensin receptor blockers, calcium channel blockers, and aspirin treatment was recorded. A history of endocarditis and the presence of coarctation of the aorta were assessed. At baseline, the symptomatic status [classified according to the New York Heart Association and Canadian Cardiovascular Society scores, as well as a history of recent syncope] was assessed. Selected patients underwent exercise testing if there was doubt about their symptomatic status.

### Echocardiography

All patients underwent baseline clinical evaluation by comprehensive M-mode and 2D echocardiography, as well as conventional and colour Doppler by experienced echocardiographers with state-of-the-art technology. Apical four-chamber and two-chamber views were used for the calculation of ventricular volumes and ejection fraction by Simpson’s biplane formula. A LVEF of ≥50% was considered normal, a LVEF of 40–50% was considered mildly depressed, a LVEF of 30–40% moderately depressed, and a LVEF of <30% severely depressed. BAV disease was confirmed in a short-axis view at the level of the aortic cusps. Aortic valve disease was quantified as mild, moderate, or severe following an integrated approach as recommended:^6^

Moderate AS was defined by an aortic valve area of 1.0–1.5 cm^2^ and a peak aortic jet velocity of 3.0–3.9 m/s. Severe AS was defined by a valve area of ≤1.0 cm^2^ and a peak aortic jet velocity of ≥4 m/s. Multiple alternative transducer positions (including the right parasternal window) were used to record peak aortic jet velocity. In cases of inconsistencies between valve areas and gradients, an integrated approach that included severity of AR, degree of valve calcification, and degree of LV hypertrophy was used to grade AS after the careful exclusion of measurement errors. Severe AR was defined by a vena contracta of >6 mm and a prominent holodiastolic flow reversal in the descending thoracic aorta and moderate AR by a vena contracta of 3–6 mm. 2D measurements of the aorta at different levels, including the aortic root, the sinotubular junction, the proximal ascending aorta, the aortic arch, and the descending aorta, were realized.

### Follow-up

All patients were followed prospectively in our HVC. Follow-up exams were scheduled every 6–24 months depending on the severity of valve lesions and aortic diameters. Events were defined as cardiac death or indication for surgery of the aortic valve and/or the ascending aorta based on current guidelines. Additional follow-up information was obtained from medical and echocardiographic records, as well as telephone interviews with the patients, their relatives, and their physicians. We obtained information about the development of cardiac symptoms, eventual surgery on the aortic valve or the ascending aorta, and death. Mortality was determined and deaths were classified as cardiac or non-cardiac. Deaths due to cardiac causes were further classified as being directly related to BAV including severe AS, AR, and/or aortic complications (sudden death or death due to congestive heart failure) or as related to other cardiac conditions. Patients were additionally stratified according to age in the following intervals: <20, 20–40, 41–60, and >60 years.

### Statistical analysis

Baseline clinical and echocardiographic characteristics and surgical data are expressed as median and quartiles (due to clearly non-normal distributions for most variables) for continuous parameters and counts and percentages for categorical variables. Median potential follow-up is calculated using the reversed Kaplan–Meier method.^7^

Event-free survival is defined as the time from the index examination in the HVC to the first of cardiac death or surgery on the aortic valve and/or the ascending aorta. Non-cardiac death is treated as a competing event for event-free survival. Overall survival is defined as the time from the index examination in the HVC to a non-cardiac or cardiac death. The magnitude of the effect of different variables at baseline (age, AR severity, AS severity, and aortic dilation) on event-free survival is graphically represented by cumulative incidence functions accounting for non-cardiac death as a competing risk. *P*-values of Gray’s test for competing risk data are reported. Overall survival of the total cohort is represented as a Kaplan–Meier curve.

The effect of each of the above-mentioned variables on event-free survival is analysed by simple Fine and Gray regression models for competing risk data. Since none of the ordinal variables showed a significant non-linear effect, a single hazard ratio [with 95% confidence intervals (CIs)] is reported for each to describe the effect comparing each category with the preceding one. Values of *P* ≤ 0.05 were considered to indicate statistical significance. All calculations were performed using SAS 9.4.

## Results

**Figure 1:**
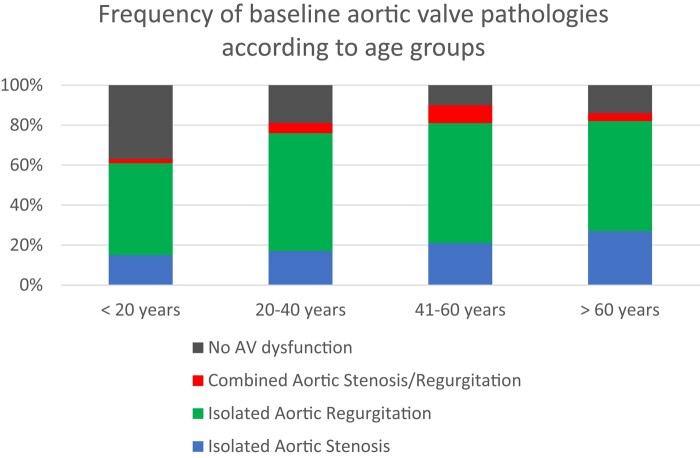
Frequency of baseline aortic valve pathologies according to age groups (<20, 20–40, 41–60, and >60 years): isolated AS (blue bars), isolated AR (green bars), combined AS/AR (red bars), and no AV dysfunction (grey bars). AV, aortic valve.

A total of 581 patients {median age 29 [interquartile range (19–43)], 157 female} were included in the study, and during a medial potential follow-up of 125 (CI 71–165) months, 158 patients developed an indication for surgery and 34 patients died. Baseline characteristics of the patients are given in *Table 1*. The majority of patients were male (*n* = 424, 73.0%). The most frequent valve dysfunction at presentation was isolated AR (*n* = 322, 55%). A total of 126 patients (22%) presented with a functionally normal aortic valve. Across all age groups, isolated AR was the most frequent aortic valve dysfunction with a prevalence between 50% and 60% (*Figure 1*). The prevalence of AS increased with age (*Figure 1*). A discordant grading of AVA and mGrad was present in 13 patients with a median age of 34 (24–44) years, 12 of which were deemed to have moderate AS, and one of them to have severe AS. These patients had a median AV-Vel of 3.8 (3.5–4) m/s, a median mGrad of 31 (29–33) mmHg, and a median AVA of 0.8 (0.7–0.9) cm^2^. Moderate AR was present in five of these patients and severe AR in two.

**Table 1:** Baseline patient characteristics of BAV patients overall and stratified according to the baseline aortic valve disease

Baseline clinical characteristics	Total population	No AV dysfunction	Isolated AS	Isolated AR	Combined AS/AR
All patients, *n* (%)	581	126 (21.7)	103 (17.7)	322 (55.4)	30 (5.2)
Sex (female), *n* (%)	157 (27.0)	46 (36.5)	43 (41.7)	62 (19.3)	6 (20.0)
Age (y), median (IQR)	29 (19–43)	20 (18–32)	32 (20–44)	30 (20–44)	39 (26–47)
Rhythm:					
Sinus rhythm, *n* (%)	561 (96.6)	121 (96.0)	100 (97.1)	312 (96.9)	28 (93.3)
A-Fib, *n* (%)	7 (1.2)	1 (0.8)	1 (1.0)	4 (1.2)	1 (3.3)
Pace maker, *n* (%)	11 (1.9)	3 (2.4)	2 (1.9)	5 (1.6)	1 (3.3)
AV junctional rhythm, *n* (%)	2 (0.3)	1 (0.8)	0 (0)	1 (0.3)	0 (0)
Risk factors at study entry:					
DM, *n* (%)	9 (1.5)	1 (0.8)	1 (1.0)	7 (2.2)	0 (0)
Hypercholesterolaemia, *n* (%)	84 (14.5)	8 (6.3)	14 (13.6)	51 (15.8)	11 (36.7)
Hypertension, *n* (%)	203 (34.9)	31 (24.6)	34 (33.0)	127 (39.4)	11 (36.7)
CAD, *n* (%)	13 (2.2)	2 (1.6)	3 (2.9)	7 (2.2)	1 (3.3)
Medication at study entry:					
Beta-blocker, *n* (%)	127 (21.9)	21 (16.7)	27 (26.2)	69 (21.4)	10 (33.3)
ACE-inhibitor, *n* (%)	111 (19.1)	13 (10.3)	18 (17.5)	73 (22.7)	7 (23.3)
AT II-blocker, *n* (%)	53 (9.1)	8 (6.3)	10 (9.7)	31 (9.6)	4 (13.3)
Statin, *n* (%)	74 (12.7)	5 (4.0)	14 (13.6)	48 (14.9)	7 (23.3)
Calcium Ch. blocker, *n* (%)	24 (4.1)	1 (0.8)	1 (1.0)	21 (6.5)	1 (3.3)
Aspirin, *n* (%)	51 (8.8)	9 (7.1)	12 (11.7)	24 (7.5)	6 (20.0)
History of endocarditis, *n* (%)	14 (2.4)	0 (0)	4 (3.9)	10 (3.1)	0 (0)
Aortic coarctation, *n* (%)	94 (16.2)	45 (35.7)	10 (9.7)	39 (12.1)	0 (0)
LV (mm), median (IQR), 574^a^	49.0 (46.0–54.0)	47.0 (44-0–50.0)	46.5 (44.0–50.0)	52.0 (47.0–57.0)	53.5 (50.0–59.0)
RV (mm), median (IQR), 573^a^	31.0 (28.0–34.0)	30.0 (28.0–34.0)	30.0 (28.0–34.0)	31.0 (28.0–34.0)	31.0 (27.0–35.0)
LA (mm), median (IQR), 575^a^	47.0 (43.0–51.0)	46.0 (43.0–50.0)	47.0 (44.0–51.0)	47.0 (43.0–51.0)	49.5 (46.0–55.0)
RA (mm), median (IQR), 574^a^	47.0 (43.0–50.0)	45.0 (42.0–49.0)	48.0 (44.0–51.0)	47.0 (43.0–50.0)	50.0 (44.0–56.0)
IVS (mm), median (IQR), 576^a^	11.0 (9.0–13.0)	9.0 (8.0–10.0)	11.5 (9.0–14.0)	11.0 (9.0–13.0)	14.5 (12.0–16.0)
Presence of aneurysm (≥40 mm), *n* (%)	190 (32.7)	25 (19.8)	30 (29.1)	119 (37.0)	16 (53.3)
Mean aortic pressure gradient (mmHg), median (IQR), 424^a^	15.0 (10.0–25.0)	11.0 (7.0–16.0)	27.0 (18.0–45.0)	12.5 (9.0–19.0)	39.5 (31.0–51.0)
Peak aortic jet velocity (m/s), median (IQR), 547^a^	2.2 (1.6–3.0)	1.9 (1.5–2.5)	3.5 (2.7–4.0)	1.9 (1.5–2.5)	4.2 (3.5–4.8)
AR pressure half time (ms), median (IQR), 72^a^	355.0 (273.0–447.0)	550.0 (555.0–555.0)	260.0 (216.0–527.0)	365.0 (290.0–429.0)	347.5 (252.5–448.0)
Aortic valve area (cm^2^), median (IQR), 100^a^	1.0 (0.8–1.2)	1.3 (1.2–2.8)	1.0 (0.8–1.1)	1.5 (1.1–2.8)	1.0 (0.8–1.2)

%, percentage within group (baseline aortic valve disease); ACE-inhibitor, angiotensin-converting-enzyme inhibitor; A-Fib, atrial fibrillation; AR, aortic regurgitation; AS, aortic stenosis; AT II-bBlocker, angiotensin II-blocker; AV, aortic valve; CAD, coronary artery disease; Calcium Ch. blocker, calcium channel blocker; *n*, number of patients; DM, diabetes mellitus; NS, non-significant; SD, standard deviation; y, years.

^a^Number of observations

A history of aortic coarctation was present in 16% of the patients.

A total of 190 (32.7%) patients presented with aortic dilatation (aortic diameter ≥ 40 mm). Aortic dilatation was more common in patients with isolated AR (119 of 322 patients; 37.0%) than in patients with isolated AS (30 of 103 patients; 29.1%). The largest aortic diameters were measured at the level of the proximal ascending aorta with 37 mm (quartiles 32–42), followed by the aortic root with 36 mm (quartiles 30–40) and the sinotubular junction with 31 mm (quartiles 27–35). Smaller diameters were measured at the level of the aortic arch with 28 mm (quartiles 24–32) and the descending aorta with 19 mm (quartiles 16–21). Fourteen (2.4%) patients had a history of infective endocarditis at study entry.

### Overall survival

**Figure 2:**
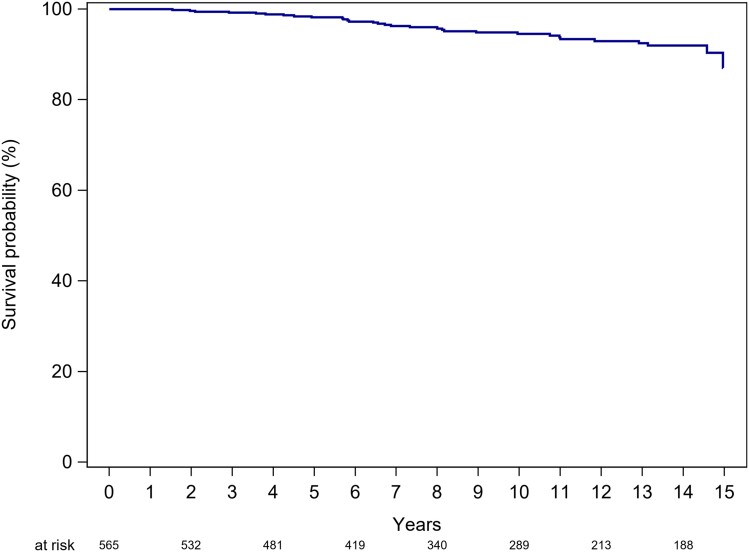
Overall survival for patients with bicuspid aortic valve disease.

The overall survival rate censored at the end of the study was 100.0% at 1 year, 99.6% (CI 98.5–99.9) at 2 years, 98.2% (CI 96.5–99.1) at 5 years, and 94.5% (CI 91.8–96.3) at 10 years (*Figure 2*).

### Event-free survival and predictors of outcome

**Figure 3:**
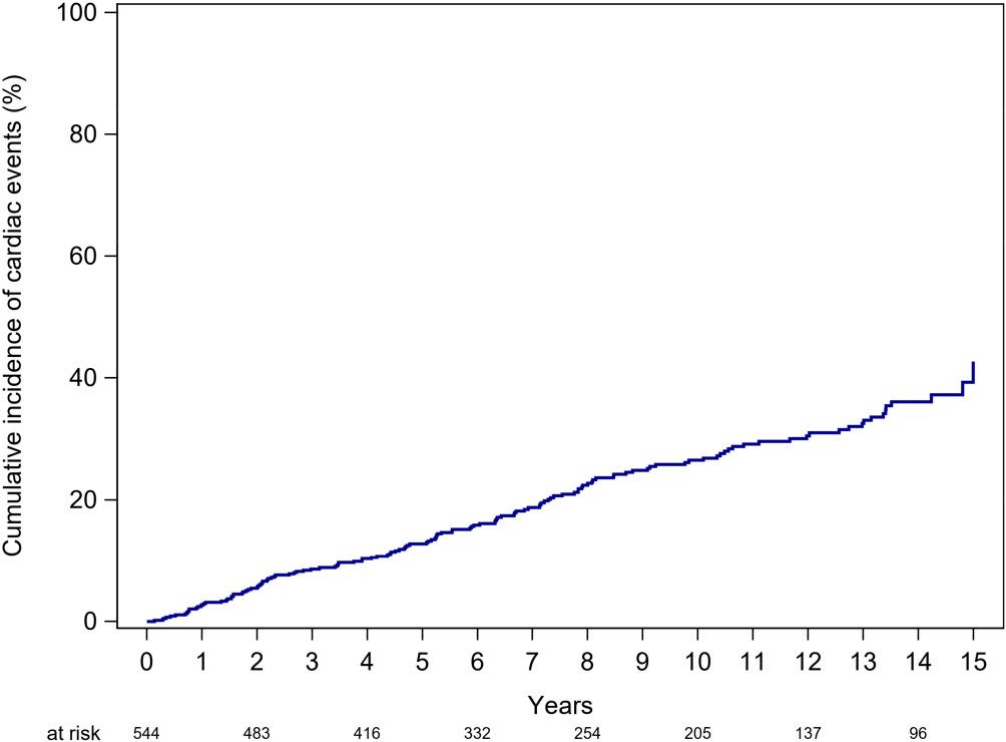
Cumulative incidence functions for cardiac events (defined as cardiac death or surgery on the aortic valve and/or the ascending aorta) for patients with bicuspid aortic valve disease.

A total of 158 patients developed an indication for surgery during follow-up; 154 of those patients eventually underwent surgery. Surgery was not performed in four patients due to patient refusal (*n* = 2), death before surgery (pulmonary embolism) (*n* = 1), and denial of surgery by the heart team because of advanced malignant disease (*n* = 1). Event-free survival rates, with events defined as surgery of the AV, the aorta, or cardiac death, were 97.2% (CI 95.5–98.4), 94.3% (CI 92.1–96.1), 87.2% (CI 84.1–90.0), and 73.5% (CI 69.1–77.7) at 1, 2, 5, and 10 years, respectively (*Figure 3*).

**Figure 4:**
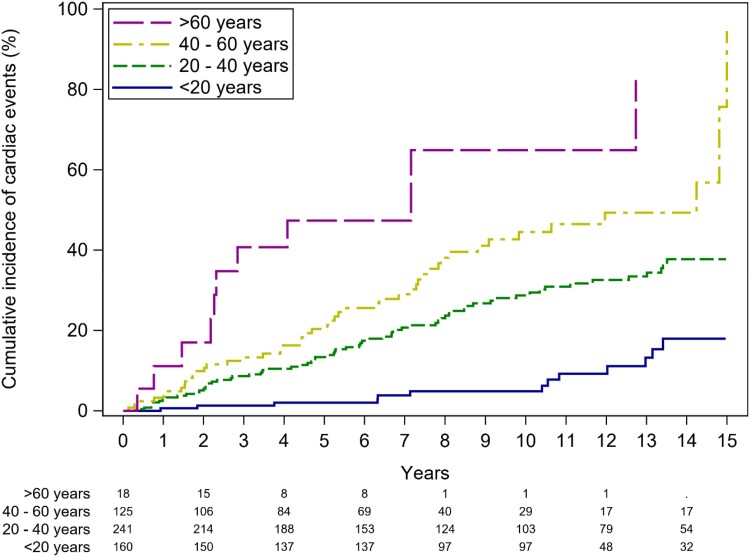
Cumulative incidence functions for cardiac events for patients with bicuspid aortic valve disease stratified according to age: <20 (blue line), 20–40 (green line), 41–60 (yellow line), and >60 (purple line).

Age was a strong predictor of events: 1-, 2-, 5-, and 10-year event-free survival rates were 99.4% (96.7–99.9), 98.7% (CI 95.7–99.7), 98.0% (CI 94.6–99.5), and 95.1% (CI 90.2–98.0) in patients < 20 years; 97.1% (CI 94.3–98.7), 94.5% (CI 91.0–96.9), 86.6% (CI 81.8–90.7), and 71.2% (CI 64.6–77.5) in patients 20–40 years; 95.9% (CI 91.3–98.5), 90.1% (CI 84.0–94.9), 79.6% (CI 71.6–86.6), and 55.5% (CI 45.1–66.4) in patients 40–60 years; and 88.9% (CI 69.6–98.3), 83.0% (CI 62.1–96.1), 52.6% (CI 30.6–78.2), and 35.1 (CI 9.8–83.5) in patients > 60 years, respectively (*P* < 0.001) (*Figure 4*).

**Figure 5:**
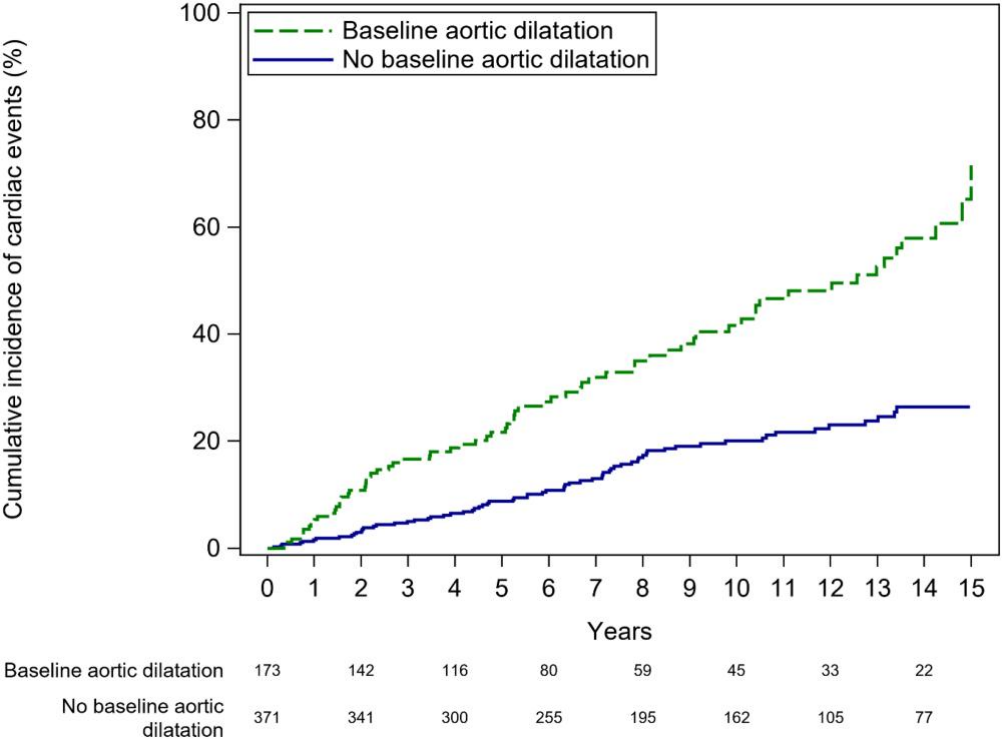
Cumulative incidence functions for cardiac events for patients with bicuspid aortic valve disease stratified according to baseline aortic diameters ≥ 40 mm (green line) versus baseline aortic diameters < 40 mm (blue line).

Also, aortic dilatation was associated with event-free survival: for patients without baseline aortic dilatation, event-free survival rates were 98.4% (CI 96.6–99.3), 96.7% (CI 94.5–98.2), 91.2% (CI 87.9–93.9), and 79.9% (CI 75.1–84.4) at 1, 2, 5, and 10 years as compared with 94.6% (CI 90.5–97.4), 89.1% (CI 83.8–93.3), 78.3% (CI 71.5–84.4), and 58.4% (CI 49.6–67.4) for patients with baseline aortic dilatation (*P* < 0.001) (*Figure 5*).

**Figure 6:**
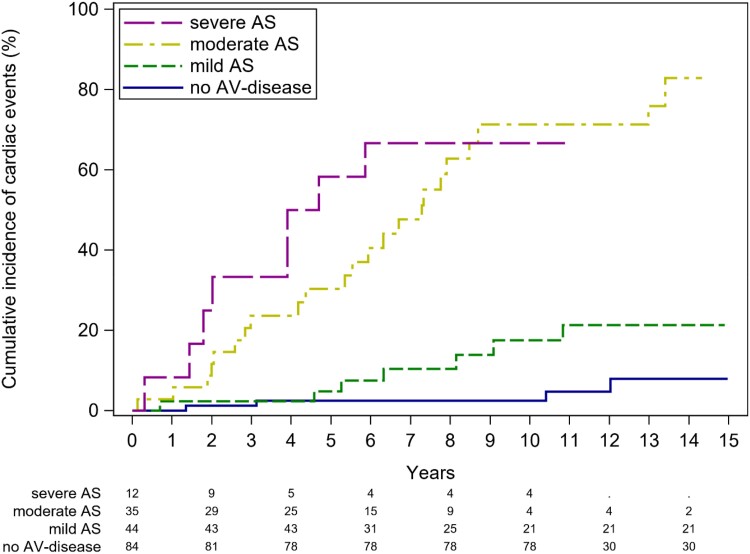
Cumulative incidence functions for cardiac events for patients with bicuspid aortic valve disease and no aortic regurgitation stratified according to the degree of severity of aortic stenosis: no aortic stenosis (blue line), mild aortic stenosis (green line), moderate aortic stenosis (yellow line), and severe aortic stenosis (purple line). AV, aortic valve.

Incremental increases in the event rate were observed with higher degrees of AS severity (*P* < 0.0001). Event-free survival according to the severity of AS after the exclusion of patients with AR is presented in *Figure 6* (*Graphical Abstract*).

**Figure 7:**
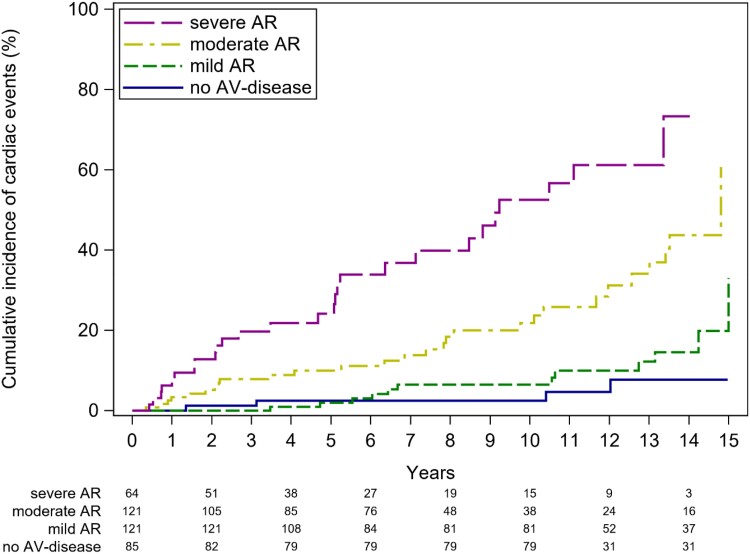
Cumulative incidence functions for cardiac events for patients with bicuspid aortic valve disease and no aortic stenosis stratified according to the degree of severity of aortic regurgitation: no aortic regurgitation (blue line), mild aortic regurgitation (green line), moderate aortic regurgitation (yellow line), and severe aortic regurgitation (purple line). AV, aortic valve.

Furthermore, event rate increased with higher degrees of AR severity (*P* < 0.0001). Event-free survival according to the severity of AR after the exclusion of patients with AS is presented in *Figure 7*.

Event-free survival differed between patients with and without a history of coarctation, with respective event-free survival rates of 97.3% (CI 95.5–98.5), 93.3% (CI 91.4–95.9), 86.2% (82.7–89.3), and 70.4% (65.4–75.2) for patients with no history of coarctation and 96.6% (91.2–99.1), 96.6% (91.2–99.1), 92.6% (85.4–97.0), 89.3% (81.0–95.1) at 1, 2, 5, and 10 years for patients with a history of coarctation (*P* = 0.0009).

In the multivariable analysis, age, AS severity, AR severity, and baseline dilation were strong independent predictors of event-free survival (*Table 2*).

**Table 2:** Potential predictors for events

Variable	Univariable analysis		Multivariable analysis	
	SHR (95% CI)	*P*-value	SHR (95% CI)	*P*-value
Age^a^	2.18 (1.79–2.66)	**<0.001**	1.33 (1.17–1.51)	**<0.001**
Baseline aortic Dilatation ≥ 40 mm^b^	2.82 (2.03–3.93)	**<0.001**	1.85 (1.31–2.63)	**0.001**
Baseline grade AS^c^	2.42 (2.00–2.94)	**<0.001**	2.31 (1.89–2.83)	**<0.001**
Baseline grade AR^c^	1.70 (1.42–2.03)	**<0.001**	1.53 (1.27–1.85)	**<0.001**

AR, aortic regurgitation; AS, aortic stenosis; CI, confidence interval; SHR, subdistribution hazard ratio.

^a^As a continuous variable (SHR refers to a 10-year increase in age).

^b^As a dichotomized variable.

^c^SHR refers to each grade (1, 2, and 3) vs. the preceding grade (linear effect).

### Mortality in patients without an indication for surgery

There were six sudden cardiac deaths in patients who did not reach a prior indication for surgery [two patients with predominant AS, two patients with combined AS and AR, one patient with predominant AR, and one patient with no AV dysfunction, all male, median age 41 (28–60) years, all with preserved LVF]. Two of these events occurred in the setting of an acute coronary syndrome in patients with previously undetected CAD and one in a patient with documented chronic ischaemic heart disease. Of the three patients without ischaemic heart disease, two underwent autopsy, where LV hypertrophy and dilatation were determined as causes of death. In one patient, no autopsy was performed, and LV dilatation was deemed cause of death by the medical examiner. Eleven non-cardiac deaths occurred in patients without indications for surgery and were due to the following reasons: amyotrophic lateral sclerosis, intracerebral haemorrhage, multiorgan dysfunction syndrome, liver cirrhosis, exacerbated inflammatory bowel disease, opioid abuse, pulmonary embolism, traffic accident (*n* = 1 for each), cancer (*n* = 3), and unknown (*n* = 1).

### Indications for surgery

**Figure 8:**
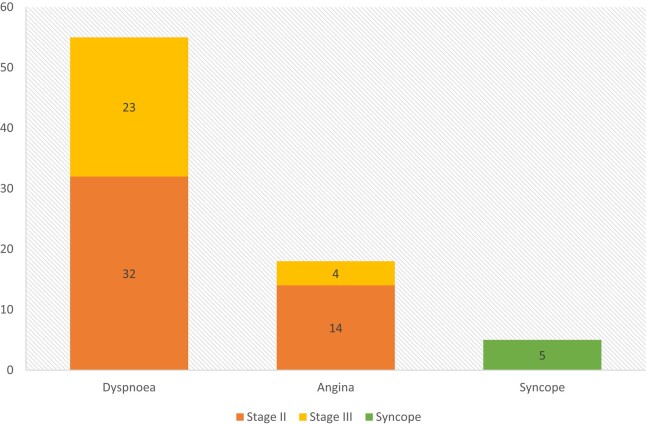
Symptoms related to bicuspid aortic valve disease.

Surgery was indicated for the following reasons: symptom onset (*n* = 78), asymptomatic left ventricular (LV) dysfunction (*n* = 22; 1 patient with predominant AS, 19 patients with predominant AR, and 2 with combined AS and AR), aortic aneurysm size meeting criteria for surgery (*n* = 22), rapid haemodynamic progression of AS^8^ (*n* = 30), infective endocarditis (*n* = 3), acute aortic dissection (*n* = 1; ascending aortic diameter at baseline 39 mm), and desire for pregnancy (two patients with asymptomatic severe AS). Of the patients having developed symptoms, the leading complaint was exertional dyspnoea (*n* = 55), followed by angina (*n* = 18), and syncope (*n* = 5) (*Figure 8*).

### Surgery

A total of 154 patients (26.5%) underwent surgery at a median age of 39.5 years (quartiles 30.0 and 50.0). Types of surgery included mechanical aortic valve replacement (AVR) (*n* = 37), mechanical AVR and reduction aortoplasty (*n* = 4), mechanical AVR and replacement of the ascending aorta (*n* = 35), Ross procedure (*n* = 23), Ross procedure and reduction aortoplasty (*n* = 6), Ross procedure and replacement of the ascending aorta (*n* = 4), biological AVR (*n* = 21), biological AVR and reduction aortoplasty (*n* = 3), biological AVR and replacement of the ascending aorta (*n* = 11), aortic valve repair (*n* = 4), isolated replacement of the ascending aorta (*n* = 3), balloon aortic valvuloplasty (*n* = 2), and heart transplantation (*n* = 1).

Concomitant mitral surgery was performed in three patients (two mechanical replacements and one repair), one patient received concomitant biological tricuspid replacement, and seven patients received concomitant aortocoronary bypass surgery. There were no peri-operative deaths.

### Post-operative survival

Three patients died in the early post-operative period (1–3 months after surgery) due to acute heart failure with LV assist device (*n* = 1) and acute myocardial infarction (*n* = 2).

## Discussion

Data on the long-term natural history and potential predictors of outcome in patients with BAV are limited.^9^ This study represents a large single-centre investigation aimed at assessing the natural course of consecutive BAV patients managed at a tertiary centre HVC.

### Main findings

Our study reveals impressive overall survival rates of 98% at 5 years and 95% at 10 years within a relatively youthful cohort, with a median age of 29 years. Of note, similar 10-year survival rates have been reported in previous studies, despite variations in recruitment periods.^10–12^ These findings provide reassurance for BAV patients regarding their life expectancy. Moreover, concerns about sudden death in the BAV patient cohort have been long-standing. In our study encompassing 581 patients, we observed six sudden deaths, two of which occurred in the context of acute coronary syndromes. This corresponds to an annualized sudden death rate of 0.12%, affirming the low risk of sudden death in BAV patients.

### Secondary findings

While mortality remained low in our study, morbidity was substantial, with 27% of patients experiencing an adverse event within 5 years, especially considering the youth of the study population. Of note, in line with previous research,^5,10–15^ a male predominance in the patient population was found, with a male-to-female ratio of 3:1.

### Predictors of cardiac events

Consistent with prior research,^11,14^ AR emerged as the most prevalent aortic valve pathology across all age groups, while the prevalence of AS increased with age.^16^ Notably, we observed the absence of an associated aortic valve pathology in 37% of patients under the age of 20 years. However, with advancing age, only a minority of patients remained free of AR or AS.

In addition to age, the baseline severity of AR and, more significantly, the degree of AS proved to be strong predictors of cardiac events, as indicated by multivariable analysis (*P* < 0.001 for both). It is worth noting that in the Olmsted County study, patients with significant aortic valve disease were excluded at baseline,^10^ and in the study by Tzemos *et al*.,^11^ the predictive role of aortic valve disease was assessed in a binary manner, distinguishing only between moderate-to-severe and no or mild disease. Our study breaks new ground by demonstrating the incremental prognostic value of mild, moderate, and severe AR and AS in relation to subsequent events. We are the first to provide event rates for patients with mild and moderate AR, as well as mild AS, which have not been described previously. Importantly, our findings reveal relatively high event rates, even in patients with moderate AS, supporting the notion of rapid disease progression linked to abnormal mechanical and shear stresses associated with BAV.^17^ Patients with a history of coarctation demonstrated higher event-free survival rates. This likely reflects that the relative majority of these patients (48%) presented with no aortic valve dysfunction and were significantly younger than patients without a history of coarctation (26.7 ± 11.2 vs. 33.2 ± 14.4 years, *P* < 0.001).

### Infective endocarditis

Concerns about the frequency of infective endocarditis in BAV patients have historically led to considerations of antibiotic prophylaxis.^18^ In contrast, some studies have reported an approximately 2% incidence of infective endocarditis, primarily affecting males.^10,11,14^ Although infective endocarditis in BAV patients is more frequently complicated by abscess formation than in tricuspid aortic valves, it does not confer higher mortality during hospitalization or at the 5-year follow-up.^19^ In our study, infective endocarditis was rare, affecting three patients and occurring at an annualized rate of 0.06%. However, it is important to note that most of these patients were included and followed before the implementation of more restrictive antibiotic prophylaxis recommendations,^20–24^ leaving uncertainties regarding current practice. Recent reports have raised awareness of higher incidences of infective endocarditis among patients with BAV.^25,26^

### Aortic complications

A significant portion of the high morbidity observed in the BAV patient cohort can be attributed to associated aortic complications.^14^ Baseline aortic dilatation, defined as aortic diameters equal to or exceeding 40 mm, was identified in 190 (32.7%) patients and emerged as a robust and independent predictor of future cardiac events. Notably, ascending aortic aneurysms constituted 14% of the surgical indications.

The risk of aortic dissection in BAV patients has been estimated to be eight times higher than in the general population, although its actual occurrence is rare, with a yearly rate of 0.03%.^5^ In our study, the risk of aortic dissection was minimal, with only one event recorded in a patient with a previous aortic diameter of 39 mm. This finding aligns with previous investigations of BAV patients,^5,14,27^ where most patients who experienced aortic dissection had thoracic aortic diameters that did not meet the criteria for elective aortic surgery as recommended by current guidelines.^28,29^ It remains uncertain whether the low incidence of dissection is due to its intrinsic rarity or reflects the effectiveness of active surveillance. Nonetheless, our study provides reassurance that current management strategies are secure, and the occurrence of aortic dissection in BAV patients is infrequent.

### Study limitations

The presence and orientation of a raphe^26^ as well as the characterization by Sievers *et al*.^30^ were not systematically assessed in our protocol and is thus not reported. However, in adult populations thus far, there is no evidence of associations between BAV phenotypes and clinical outcomes^5,10,11^

The present study was not designed to analyse the precise progression of AS, AR, and aortic dilatation but rather the incidence of clinical aortic valve and ascending aortic complications highlighting the importance of close clinical follow-up and implementation of current guidelines.

Maximal aortic diameter measurements were not available in certain patients, particularly those with echocardiographic data obtained years prior. However, despite this constraint, the study retained the ability to categorize patients based on the presence or absence of aneurysm (<40 mm or not).

Selection bias might be present in this study population of patients referred to a tertiary centre.

## Conclusion

BAV disease is associated with a high rate of cardiac events, but state-of-the-art care results in good survival with low rates of infective endocarditis, aortic dissection, and sudden death. Incremental degrees of AR and AS at baseline across the entire spectrum of severity, the presence of aortic dilatation, and age are predictive of cardiac events.

## Data Availability

The data underlying this article will be shared on reasonable request to the corresponding author.
